# Prevalence and Associated Factors with Frailty Using the Kihon Checklist among Community-Dwelling Older Adults in Taiwan

**DOI:** 10.3390/medicina60081231

**Published:** 2024-07-29

**Authors:** Chien-Chih Chen, Wei-Chien Hsu, Yi-Hsuan Wu, Fang-Yu Lai, Pei-Yu Yang, I-Ching Lin

**Affiliations:** 1 Department of Family Medicine, Asia University Hospital, No. 222, FuSin Rd., Wufeng, Taichung 41354, Taiwan; D51120@auh.org.tw (C.-C.C.); D51121@auh.org.tw (W.-C.H.); 018486@tool.caaumed.org.tw (Y.-H.W.); 180016@tool.caaumed.org.tw (F.-Y.L.); 2 Department of Medical Technology, Jenteh Junior College of Medicine, Nursing and Management, No. 79-9 Sha-Luen Hu, Xi-Zhou Li, Hou-Loung Town, Miaoli County 35664, Taiwan; 3 Department of Kinesiology, Health and Leisure, Chienkuo Technology University, No. 1, Chiehshou North Road, Changhua 50000, Taiwan; 4 Department of Healthcare Administration, Asia University, No. 500, Lioufeng Rd., Wufeng, Taichung 41354, Taiwan

**Keywords:** frailty, physical fitness indicators, Kihon Checklist, community-dwelling older adult

## Abstract

*Background and Objectives*: Frailty in older adults is associated with adverse health outcomes. This study aimed to analyze the frailty status of community-dwelling older adults in Taiwan using the Kihon Checklist (KCL) and explore associations with demographic, physiological, and functional factors. *Materials and Methods*: In this cross-sectional study, 278 community-dwelling older adults were classified as robust, prefrail, or frail based on their KCL scores. Participants underwent physical fitness assessments including muscle strength and endurance tests, walking speed tests, and flexibility tests. One-way ANOVA and logistic regression analyses were used to examine differences and associations between frailty status and physical fitness indicators. *Results*: 36% of participants were robust, 47.1% prefrail, and 16.9% frail. The robust group significantly outperformed the prefrail and frail groups in the 30 s sit-to-stand test, 2.44 m sit-to-walk test, and walking speed (*p* < 0.001). The 2.44 m sit-to-walk test was a significant predictor of prefrailty (OR = 1.18, 95% CI = 1.02–1.36) after adjusting for other physical fitness indicators. *Conclusions*: Lower limb functional capacity, particularly in the 2.44 m sit-to-walk test, was significantly associated with pre-frailty among community-dwelling older adults in Taiwan. Early screening, the classification of frailty by the Kihon Checklist, and targeted interventions focusing on lower limb strength, endurance, and mobility are crucial for preventing and delaying frailty progression in older populations.

## 1. Introduction

The rapid growth of the aging population is a major public health challenge globally. With the shift in demographics towards an older populace, the geriatric syndrome of frailty is becoming an increasingly important issue. Frailty in older adults refers to a state of diminished physiological reserve, and it is associated with an increased risk of adverse health outcomes such as falls, disability, hospitalization, and mortality [1,2]. A range of studies have reported varying prevalence rates of frailty in the global population. In a systematic review and meta-analysis, O’Caoimh et al. found that the prevalence of frailty ranged from 7% to 24%, with higher rates in studies using the deficit accumulation model [3]. In addition, Farooqi et al. reported that when using global thresholds to define physical frailty, the prevalence ranged from a low of 2.4% in North America/Europe to a high of 20.1% in Africa. However, when using region-specific thresholds, the prevalence ranged from a low of 4.1% in Russia/Central Asia to a high of 8.8% in the Middle East, with regional variations being much smaller [4]. Effective prevention and management of frailty can not only improve the quality of life for older individuals but also substantially reduce the enormous related healthcare and caregiving costs.

Taiwan has already transitioned into an aged society, with elderly individuals aged 65 and above accounting for 17.6% of the total population in 2022 [5]. Therefore, the early detection and prevention of frailty among community-dwelling older adults is crucial for maintaining their health and delaying the progression of functional decline. Currently, various assessment tools such as the Taiwanese Frailty Evaluation Scale and Fried Frailty Criteria are available for screening frailty. Among them, the Japanese Kihon Checklist (KCL) is a simple yet reliable and valid instrument for rapidly assessing the risk of frailty in older individuals [6,7].

Kihon Checklist has been translated into a Chinese version, and it demonstrated good internal consistency, excellent test–retest reliability, and acceptable construct validity, with moderate to low correlations with other established scales. The KCL Chinese version exhibited excellent discrimination with high sensitivity and specificity, making it a promising and convenient screening tool for frailty in elderly populations [8]. The Kihon Checklist has demonstrated its versatility and applicability across different cultural contexts. Studies validating the KCL in various countries have consistently shown its reliability and validity as a frailty screening tool. In Turkey, Esenkaya et al. found that the KCL demonstrated good internal consistency (Cronbach’s α = 0.876) and high sensitivity and specificity for detecting frailty among Turkish older adults [9]. Similarly, the Brazilian Portuguese version of the KCL showed a strong correlation with the original Japanese version (r = 0.764) and good internal consistency (Cronbach’s α = 0.787) [10], as reported by Sewo Sampaio et al. In Spain, Sentandreu-Mañó et al. validated both the original 25-item and a reduced 15-item version of the KCL, finding adequate internal consistency and validity, particularly for the 15-item version [11]. These studies collectively highlight the KCL’s robustness as a frailty screening tool across different cultural and linguistic settings. The consistent performance of the KCL across these diverse populations underscores its potential as a universally applicable instrument for frailty assessment. Our study’s findings in the Taiwanese context further contribute to this growing body of evidence, supporting the KCL’s utility in Asian populations beyond its original Japanese setting. This cross-cultural validity makes the KCL a valuable tool for international comparisons of frailty prevalence and characteristics, potentially facilitating more standardized approaches to frailty research and intervention globally.

This study aimed to use the KCL and a series of physical fitness assessments to analyze the frailty status of community-dwelling older adults in Taiwan and explore the associations with demographic, physiological, and functional factors. By gaining insights into the key determinants of frailty, we hope to provide an evidence base for developing effective health promotion strategies and exercise interventions to delay the progression of frailty among the aging population in Taiwan, ultimately promoting their physical and mental well-being.

## 2. Materials and Methods

**Figure 1:**
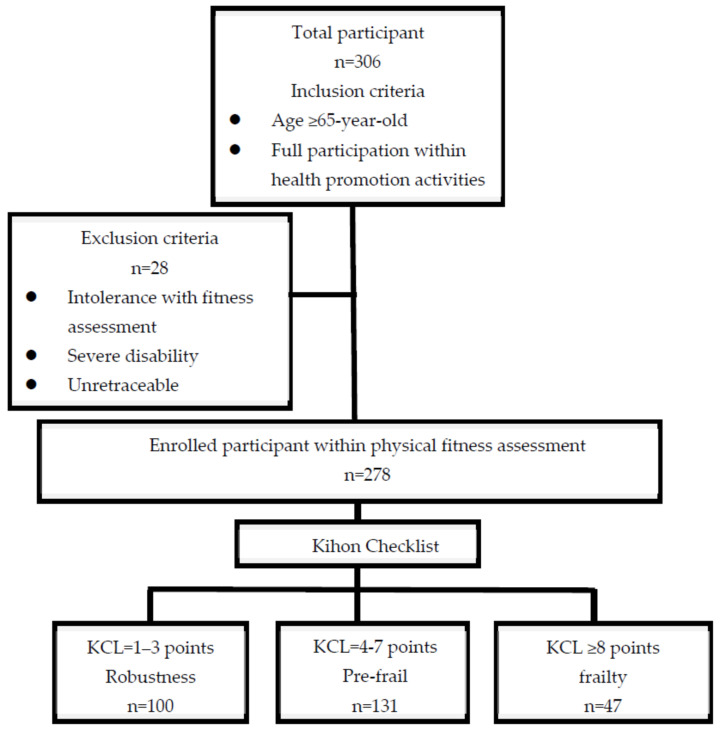
Study enrollment flow chart.

The Prevention and Community Medical Center at Asia University Hospital (Taichung, Taiwan) began enrolling participants from community health hubs for a generational study in August 2022. The initial enrollment included 306 volunteers aged 65 years or older who participated in health promotion activities, including health education lectures, exercise programs, and physical fitness assessments. (Figure 1)

The inclusion criteria were (1) Age 65 years or older (2) Ability to participate in health promotion activities (3) Willingness to undergo physical fitness assessments.

Exclusion criteria were (1) Severe disability preventing participation in physical fitness tests (2) Inability to be contacted or followed up (3) Lack of cooperation with physical fitness assessments.

After applying these criteria, 28 participants were excluded. The final sample consisted of 278 community-dwelling older adults who met all inclusion criteria and completed the necessary assessments.

All participants provided informed consent before enrollment in this study. The study protocol was approved by the Institutional Review Board of China Medical University & Hospital (approval number: CMUH111-REC3-110).

Our study examined frailty status as the primary dependent variable, categorized as robust, prefrail, or frail based on Kihon Checklist (KCL) scores. Independent variables included demographic characteristics (age, gender), anthropometric measurements (height, weight, BMI, waist circumference), and various physical fitness indicators. Lower body function was assessed through sit-to-stand tests and walking speed tests, while upper body strength was measured by bicep curls and grip strength. Flexibility was evaluated using upper back scratch and seated forward bend tests. Body composition variables included body fat percentage, skeletal muscle percentage, visceral fat percentage, bone mineral density, and calf circumference. Body composition was checked by Omron body fat monitor, HBF-370, Manufactured by Krell Precisionco., Ltd., Yangzhou, China. Bone density was checked by “Hologic” Sahara Clinical Bone Sonometer, Hologic, Inc., Bedford, MA, USA. Physiological parameters such as blood pressure were also recorded. (HEM-7121, MVN0032000 Omron Healthcare Manufacturing Vietnam Co., Ltd. Thu Dau Mot City, Binh Duong Province, Vietnam) Additionally, sarcopenia status was diagnosed based on the 2019 Asian Working Group for Sarcopenia criteria. These variables were selected for their potential association with frailty status, as indicated by previous literature and our research objectives, providing a comprehensive assessment of participants’ functional and physiological status in relation to frailty classification. Comprehensive tests were administered to obtain the participants’ overall functional capacity and mobility conditions, as described below.

### 2.1. Muscle Strength and Endurance Tests

- The 30 s Sit-to-Stand Test: The participants sat in a chair with their arms crossed at the chest, and the number of full sit-to-stand cycles completed within 30 s was recorded to assess lower body muscle strength and endurance.
- Five Times Sit-to-Stand Test: The participants rose from a 40 cm high chair to a full standing position 5 times, and the time taken to complete the task was recorded to evaluate lower body muscle strength.
- Number of Bicep Curls in 30 s: With the participants seated and holding a dumbbell (8 lbs for men, 5 lbs for women), the number of correct bicep curls performed within 30 s was recorded to assess upper body muscle endurance.
- Maximum Grip Strength Test: Using a hand dynamometer (Model EH101, Zhongshan Camry Electronic Co. Ltd.: Zhongshan, China: 90 kg capacity, 356 g weight), the maximum grip strength was measured twice for each hand, and the highest value was recorded to evaluate upper body muscle strength.

### 2.2. Walking Speed Tests

- The 2.44 m Sit-to-Walk Test: The participants rose from a chair, walked around a designated obstacle, and returned to a seated position, and the time taken to complete the course was recorded to assess lower body mobility.
- The 4 m Walking Speed Test: The participants were allowed to use an assistive device and instructed to walk at their normal pace for 4 m, and the faster of two trials was recorded to evaluate lower body mobility.

### 2.3. Flexibility Tests

- Upper Back Scratch Test: With one hand reaching over the shoulder and the other reaching up the back, the distance between the middle fingertips was measured to assess upper body flexibility.
- Seated Forward Bend Test: With one leg extended in a seated position, the participants reached towards their toes with overlapping hands, and the ability to reach beyond their toes was recorded to assess lower body flexibility.

### 2.4. Kihon Checklist (KCL)

The research team used the KCL Chinese version as a rapid screening tool to evaluate the risk of frailty among the elderly participants. The KCL is a comprehensive 25-item assessment tool that can be used to identify older adults at high risk of frailty. Participants with a total KCL score below 3 were categorized as robust, those with a score between 4 and 7 were considered prefrail, and those with a score exceeding 7 as frail [12,13,14]. We examined differences in physical fitness performance and physiological parameters across these frailty subgroups, with the aim of providing insights to guide subsequent intervention strategies.

### 2.5. Diagnosis of Sarcopenia

For the diagnosis of sarcopenia among the enrolled participants, the 2019 Asian Working Group for Sarcopenia (AWGS) consensus diagnostic criteria were applied [15]. Participants were classified as having sarcopenia if their grip strength was below 28 kg for men and 18 kg for women, in combination with a bioelectrical impedance analysis (BIA) measured skeletal muscle mass index (SMI) of less than 7.0 kg/m^2^ for men and 5.7 kg/m^2^ for women. Moreover, if participants met the aforementioned diagnostic criteria and took more than 12 s to complete the five times sit-to-stand test, they were further categorized as having severe sarcopenia.

### 2.6. Statistical Analysis

All statistical analyses were performed using IBM SPSS Statistics, version 25, IBM Corp., Armonk, NY, USA. Descriptive statistics were calculated for all variables, with continuous data presented as mean ± standard deviation and categorical data as frequencies and percentages.

One-way ANOVA was used to compare physical fitness indicators and physiological parameters across the three frailty groups (robust, prefrail, and frail). Post hoc analyses were conducted using Tukey’s HSD test for pairwise comparisons when ANOVA results were significant.

Binary logistic regression analyses were performed to identify physical fitness indicators associated with prefrailty. Three models were constructed: a crude model, an adjusted model including only physical fitness indicators, and a fully adjusted model incorporating age and body fat percentage. Odds ratios (OR) with 95% confidence intervals (CI) were calculated.

Chi-square tests were used for categorical variables. Statistical significance was set at *p* < 0.05 for all analyses.

## 3. Results

A total of 278 participants were included in the analysis of this generational study. Based on KCL scores, 100 (36%) participants were classified as robust, 131 (47.1%) as pre-frail, and 47 (16.9%) as frail. One-way ANOVA revealed a significantly higher average age in the frail group (79.68 ± 5.86 years) compared with the prefrail (78.31 ± 5.92 years) and robust (75.59 ± 6.16 years) groups (*p* < 0.001). However, there were no significant differences among the three groups in systolic and diastolic blood pressure, height, weight, body mass index, and waist circumference.

One-way ANOVA of the physical fitness tests showed that the robust group outperformed the pre-frail and frail groups in the 30 s sit-to-stand test, 2.44 m sit-to-walk test, and walking speed (*p* < 0.001) The prefrail group exhibited superior walking speed compared with the frail group, with no significant differences in other physical fitness test results. In terms of body composition, the three groups differed significantly in body fat percentage, with the robust group having a lower body fat percentage and the frail group having a significantly higher average body fat percentage (*p* = 0.019). No significant differences were observed among the three groups in bone density, grip strength, relative grip strength, calf circumference, skeletal muscle percentage, and visceral fat percentage (Table 1).

Using binary logistic regression analysis, the 30 s sit-to-stand test (odds ratio [OR] = 0.90, 95% confidence interval [CI] = 0.85–0.95), 2.44 m sit-to-walk test (OR = 1.29, 95% CI = 1.15–1.45), and walking speed (OR = 0.09, 95% CI = 0.29–0.279) in the crude model were significant predictors of prefrailty (Table 2). However, in the adjusted model, only the 2.44 m sit-to-walk test (OR = 1.18, 95% CI = 1.02–1.36) remained significantly associated with prefrailty. When age and body fat were included in the adjusted model (Model 2), the 30 s sit-to-stand test (OR = 0.96, 95% CI = 0.90–1.03), 2.44 m sit-to-walk test (OR = 1.14, 95% CI = 0.98–1.33), and walking speed (OR = 0.54, 95% CI = 0.12–2.40) lost their predictive abilities.

## 4. Discussion

The results of this study reveal a significant association between lower limb functional capacity and prefrailty among community-dwelling older adults in Taiwan. The findings emphasize the crucial role of lower limb muscle strength, endurance, and walking speed in distinguishing between different levels of frailty. Participants in the robust group demonstrated markedly superior performance in the 30 s sit-to-stand test, 2.44 m sit-to-walk test, and walking speed compared with their counterparts in the prefrail and frail groups. These results suggest that lower limb function may serve as a pivotal indicator in assessing the degree of frailty in older individuals. Batista et al. found that lower limb muscle strength was significantly associated with frailty in the elderly [16], and their results were further supported by Shah et al., who found a moderate correlation between frailty and functional lower extremity strength in community-dwelling older adults [17]. This relationship was confirmed by another study which extended the findings to cognitive frailty, with lower extremity function tests such as the 6 min walk test, timed up and go test, and five times sit-to-stand test being potential predictors of cognitive frailty [18]. These findings collectively suggest that lower limb function, including muscle strength and endurance, may play a crucial role in the development and progression of frailty in the elderly.

In this study, we used two different assessment tools to evaluate the walking speed of the participants. In particular, the 2.44 m sit-to-walk test was a stronger predictor of prefrailty physical fitness performance. In addition, walking speed was a significant predictor of frailty levels according to the KCL criteria. Walking speed <0.8 m/s has been suggested to be an early indicator of frailty among older adults [19]. In addition, Ritt et al. reported that reduced walking speed was significantly associated with frailty status across different frailty measures [20].

According to adjusted model 2 in this study, which incorporated age and body composition, the predictive ability of the physical fitness indicators for prefrailty may have been attenuated. Recent studies have consistently shown that aging is also associated with a decline in walking speed and that this decline is influenced by a range of factors including subjective age [21], lower extremity impairment, and physical inactivity. However, several studies demonstrated that leg extensor power, standing balance, and physical activity were the most significant factors associated with this decline in older community-dwelling adults [22,23]. These studies illustrate that aging can significantly impact walking speed, lower limb muscle strength, and standing balance. As individuals grow older, their physical capacity naturally declines, which may confound the associations observed between prefrailty and various measures of functional performance. Consequently, age serves as a crucial covariate when examining the relationship between prefrailty and physical fitness indicators.

Our study contributes to the global research landscape on frailty prevention and management among older adults, notably by being the first to comprehensively examine the relationship between physical fitness indicators and KCL scores in Taiwan. While international studies have extensively explored frailty, the relationship between comprehensive physical fitness assessments and the KCL has remained largely unexplored in Taiwan. Our research aligns with and extends the findings of several key international studies. For instance, Imai et al.’s work on factors maintaining physical function in Japan underscores the importance of early intervention [6], a principle our study reinforces in the Taiwanese population. Tsuji et al.’s study revealed that participation in specific types of group sports and exercises, such as hiking, walking, and tennis, was associated with significant prevention of frailty score increases over a 3-year period. Their findings demonstrated that the effectiveness of group activities in preventing frailty varies depending on the type of sport or exercise, with some activities showing stronger associations with frailty prevention for both men and women [24] Our study mirrors Ohashi et al.’s five-year study on frailty prevalence using the KCL in Japan, enabling valuable cross-cultural comparisons and validating the KCL’s applicability across different Asian populations [25]. Furthermore, Hayashi et al.’s research on community-based group resistance exercises in Japan resonates with our findings, particularly in demonstrating how sustained physical activity engagement can delay frailty onset [26]. Uniquely, our study is the first in Taiwan to comprehensively examine the correlation between a wide range of physical fitness assessments and KCL scores. This novel approach not only fills a critical research gap in Taiwan but also enriches the global understanding of frailty prevention strategies. By providing Taiwan-specific insights and integrating them with international findings, our research contributes to a more nuanced, culturally informed approach to frailty prevention and management in diverse aging populations.

The physical fitness indicators associated with each frailty group identified in this study not only provide insights into the characteristics of frailty but also offer valuable metrics for monitoring the outcomes of interventions, particularly exercise-based programs. These indicators, such as the 30 s sit-to-stand test, 2.44 m sit-to-walk test, and walking speed, can serve as practical and measurable outcomes to assess the effectiveness of exercise interventions aimed at preventing or mitigating frailty. By regularly evaluating these parameters, healthcare providers and researchers can track the progress of individuals transitioning between frailty categories and adjust interventions accordingly. This approach allows for a more personalized and targeted strategy in managing frailty among community-dwelling older adults.

Exercise interventions have shown significant promise in improving physical function and delaying or even reversing frailty in older adults. Resistance training, in particular, has emerged as an effective strategy to enhance muscle strength, physical fitness, and metabolic health in prefrail and frail elderly populations. The studies by Lai et al. and Talar et al. demonstrate that resistance exercise programs can lead to improvements in lower limb muscle strength, walking speed, balance, and overall physical performance [27,28]. Furthermore, as shown by Lai et al.’s dose-response study, both the intensity and volume of resistance training play crucial roles in determining the magnitude of these benefits [29]. Our research builds upon these findings by examining the relationship between physical fitness indicators and frailty classification using the Kihon Checklist in Taiwanese older adults. By identifying specific physical fitness parameters strongly associated with prefrailty and frailty, our study provides valuable insights for designing targeted exercise interventions. These results can guide healthcare professionals in developing more effective, personalized resistance training programs that address the unique needs of prefrail and frail older adults. Future interventions based on our findings may focus on improving key indicators such as lower limb strength and gait speed, potentially leading to more efficient strategies for preventing and managing frailty in the aging population.

This study has several limitations. First, the findings may be limited due to the sample characteristics. As participants were recruited from community health hubs, they may represent a relatively healthy and motivated subgroup of the elderly population. Future research should aim to include a more diverse sample that better represents the broader spectrum of older adults in Taiwan. Second, the cross-sectional design of the study precludes any causal inferences about the relationships among lower limb function, walking speed, and frailty status. Longitudinal studies are needed to explain the temporal sequence and potential bidirectional associations among these variables.

Despite these limitations, our findings provide valuable insights into the importance of lower limb function and walking speed in the context of assessing frailty among community-dwelling older adults in Taiwan. Our findings further underscore the need for early screening and targeted interventions to maintain functional capacity and prevent or delay the progression of frailty. Researchers, healthcare professionals, and policymakers should prioritize the development and implementation of effective health promotion strategies that focus on enhancing lower limb strength, endurance, and mobility in older adults, ultimately promoting their overall well-being and quality of life.

## 5. Conclusions

In this preliminary investigation, we found a significant correlation between perceived frailty among community-dwelling older adults and lower limb functional capacity. The results highlight the importance of lower limb function in distinguishing between different frailty levels, as the robust group outperformed the prefrail and frail groups in the 30 s sit-to-stand test, 2.44 m sit-to-walk test, and walking speed. The current study holds significant importance in the field of geriatric research, particularly in the context of the rapidly aging population in Taiwan. By employing a comprehensive approach combining the KCL and a diverse array of physical fitness assessments, this study provides a unique and multidimensional perspective on frailty among community-dwelling older adults. Focusing on early detection, comprehensive assessments, and personalized interventions that promote physical function and overall well-being can help to achieve healthy aging and improve the quality of life for older adults in Taiwan and beyond. This study serves as a foundation for future research and underscores the importance of adopting a holistic, multidisciplinary approach to understanding and managing frailty in the elderly population.

## Figures and Tables

**Table 1:** Characteristics of the participants.

	Robust Group (KCL ≤ 3)	Prefrail Group (KCL 4–7)	Frail Group (KCL ≥ 8)	
Total	100 (36%)	131 (47.1%)	47 (16.9%)	278 (100%)
Gender	Male: 19 (19%) Female: 81 (81%)	Male: 26 (19.8%) Female: 105 (80.2%)	Male: 4 (8.5%) Female: 43 (91.5%)	*p* = 0.195
Age (years)	75.59 ± 6.16	78.31 ± 5.92	79.68 ± 5.86	*p* < 0.001
SBP (mmHg)	127.62 ± 15.24	126.79 ± 15.81	124.45 ± 11.35	*p* = 0.485
DBP (mmHg)	68.99 ± 11.02	68.44 ± 10.68	66.32 ± 10.05	*p* = 0.361
Weight (kg)	58.93 ± 9.63	59.20 ± 10.56	59.01 ± 9.36	*p* = 0.979
Height (cm)	155.10 ± 7.36	154.10 ± 7.54	154.27 ± 7.24	*p* = 0.585
BMI	24.49 ± 3.56	24.70 ± 4.64	24.78 ± 3.34	*p* = 0.892
Waistline (cm)	83.33 ± 9.15	83.56 ± 10.31	83.11 ± 9.62	*p* = 0.636
**Muscle strength and endurance**				
30 s sit-to-stand test (seconds)	15.97 ± 4.15	14.21 ± 4.61	13.04 ± 4.71	*p* < 0.001
5 times sit-to-stand test (seconds)	10.36 ± 3.09	10.69 ± 3.89	11.91 ± 4.97	*p* = 0.069
Number of bicep curls in 30 s	19.88 ± 6.48	20.00 ± 5.06	18.42 ± 5.27	*p* = 0.236
Grip strength (maximum) (kg)	22.57 ± 6.20	22.81 ± 7.49	20.33 ± 8.07	*p* = 0.114
Relative grip strength: Grip strength/weight	0.386 ± 0.109	0.392 ± 0.126	0.348 ± 0.122	*p* = 0.096
**Walk speed**				
2.44 m sit-to-walk test (seconds)	8.23 ± 2.21	9.44 ± 2.52	10.28 ± 2.73	*p* < 0.001
4 m walking speed test (seconds)	4.26 ± 1.06	4.73 ± 1.22	5.33 ± 1.61	*p* < 0.001
Walking speed (m/s) *	0.99 ± 0.24	0.88 ± 0.23	0.81 ± 0.22	*p* < 0.001
**Flexibility**				
Upper extremities (upper back scratch test)	−12.96 ± 16.38	−12.73 ± 15.01	−18.25 ± 16.99	*p* = 0.103
Lower extremities (seated forward bend test)	0.68 ± 7.31	−0.78 ± 8.26	−1.26 ± 8.54	*p* = 0.267
**Body composition**				
Bone mineral density	−1.97 ± 0.86	−2.08 ± 0.87	−2.02 ± 1.15	*p* = 0.663
Calf circumference (average) (cm)	31.89 ± 4.35	31.98 ± 3.15	31.97 ± 3.08	*p* = 0.98
Body fat percentage (%)	34.37 ± 5.57	36.08 ± 5.07	36.42 ± 4.41	*p* = 0.019
Visceral fat percentage (%)	9.83 ± 4.87	10.58 ± 5.56	10.40 ± 4.76	*p* = 0.545
Skeletal muscle percentage (%)	22.92 ± 2.71	22.23 ± 2.83	21.89 ± 2.43	*p* = 0.057
SMI	5.65 ± 1.05	5.50 ± 1.09	5.40 ± 1.06	*p* = 0.344
**Diagnosis of sarcopenia**				
Sarcopenia	32 (32%)	51 (38.9%)	24 (51.1%)	*p* = 0.085
Sarcopenia level	Nonsarcopenia: 68 (68%) Sarcopenia: 26 (26%) Severe sarcopenia: 6 (6%)	Nonsarcopenia: 80 (61.1%) Sarcopenia: 44 (33.6%) Severe sarcopenia: 7 (5.3%)	Nonsarcopenia: 23 (48.9%) Sarcopenia: 17 (36.2%) Severe sarcopenia: 7 (14.9%)	*p* = 0.089

Abbreviations: KCL: Kihon checklist; SBP: systolic blood pressure; DBP: diastolic blood pressure; BMI: body mass index; SMI: skeletal muscle index. * The value was derived from the 4-m walking speed test.

**Table 2:** Logistic regression analysis for factors associated with prefrailty.

Crude Model			
	Odds Ratio	95% CI	*p*-Value
30 s sit-to-stand test	0.899	0.847–0.954	<0.001
2.44 m sit-to-walk test	1.293	1.150–1.454	<0.001
Walking speed	0.090	0.029–0.279	<0.001
Adjusted Model 1 *			
	**Odds Ratio**	**95% CI**	***p*** **-Value**
30 s sit-to-stand test	0.961	0.898–1.029	0.255
2.44 m sit-to-walk test	1.175	1.016–1.359	0.03
Walking speed	0.372	0.088–1.584	0.181
Adjusted Model 2 **			
	**Odds Ratio**	**95% CI**	***p*** **-Value**
30 s sit-to-stand test	0.961	0.897–1.031	0.961
2.44 m sit-to-walk test	1.141	0.981–1.326	0.087
Walking speed	0.542	0.122–2.407	0.421

* Model 1 was adjusted for 30 s sit-to-stand test, 2.44 m sit-to-walk test, and walking speed. ** Model 2 was adjusted for 30 s sit-to-stand test, 2.44 m sit-to-walk test, walking speed, age, and body fat percentage.

## Data Availability

Participants were enrolled from the Asia University Hospital health examination center. The data presented in our study are available on request from the first and corresponding author. The data are not publicly available due to limitations in obtaining approval from the IRB for the disclosure of data. If anyone requires the data of this study, please contact the corresponding author.
